# Landscape-scale variation in the canopy mycobiome in temperate beech and spruce forest stands explained by leaf water content and elevation

**DOI:** 10.1007/s10342-025-01768-3

**Published:** 2025-03-29

**Authors:** Yiwei Duan, Andjin Siegenthaler, Andrew K. Skidmore, Marco Heurich, Haidi Abdullah, Anthony A. Chariton, Ivo Laros, Mélody Rousseau, G. Arjen de Groot

**Affiliations:** 1 Faculty of Geo-Information Science and Earth Observation (ITC), University of Twente, Drienerlolaan 5, PO Box 217, 7500 AE Enschede, The Netherlands; 2 Department of National Park Monitoring and Animal Management, Bavarian Forest National Park, Grafenau, Germany; 3 School of Natural Sciences, Macquarie University, Sydney, NSW Australia; 4 Wageningen Environmental Research, Wageningen UR, P.O. Box 46, 6700 AA Wageningen, The Netherlands; 5 Faculty of Environment and Natural Resources, University of Freiburg, Freiburg im Breisgau, Germany; 6 Institute for Forest and Wildlife Management, University of Inland Norway, Koppang, Norway

**Keywords:** Phyllosphere, Microbiome, ITS, European forests, *Fagus sylvatica*, *Picea abies*

## Abstract

Fungi represent a significant portion of Earth's biological diversity and are essential for ecosystem functions like organic matter decomposition and nutrient cycling. While fungi associated with plant roots have been extensively studied, our understanding of fungi in the forest canopies remains limited. To investigate the landscape-scale variation in the canopy mycobiome of temperate beech and spruce forest stands in the Bavarian Forest National Park (Germany), we examined the influence of geophysical conditions and host traits. We found that elevation significantly influenced fungal diversity and composition, with distinct effects observed in both beech and spruce stands. Moreover, canopy water content, a key indicator of tree vitality, was also strongly associated with changes in the canopy fungi community, suggesting a potential link between forest water stress and the forest canopy mycobiome. Our differential abundance analysis further identified a total of 41 fungal families as potential bioindicators: 17 families in beech stands and 9 in spruce stands were significantly associated with elevation, while 9 families in beech stands and 6 in spruce stands were linked to variations in leaf water content. These findings enhance our understanding of the spatial patterns of forest canopy microbial biodiversity and species distributions.

**Supplementary Information:**

The online version contains supplementary material available at 10.1007/s10342-025-01768-3.

## Introduction

Fungi represent a large proportion of the biological diversity on Earth (Peay et al. 2016) and are indispensable for various ecosystem functions such as organic matter decomposition (van der Wal et al. 2013) and nutrient cycling (Read and Perez‐Moreno 2003). Plant-associated fungi form intimate relationships with their plant hosts, establishing symbiotic relationships that can be mutualistic, parasitic, or commensal (Zeilinger et al. 2016). These interactions can influence the health and survival of the host plants. (Gupta et al. 2021; Horbach et al. 2011). Despite extensive research on root-associated fungi (Toju et al. 2013; Bonfante and Genre 2010), our understanding of leaf-associated fungi remains limited. This gap in knowledge is particularly critical in forests, where the above-ground portions of plants are estimated to constitute 78% of the total forest biomass globally (Ma et al. 2021). Forest canopies offer a unique habitat to various organisms (Vieira and Monteiro‐Filho 2003). These canopies are classified as extreme environments due to constant temperature oscillations, strong UV light, and fluctuating water conditions. Identifying the variables and processes that shape the canopy mycobiomes is essential for understanding host plants’ response to a changing environment, including climate-driven stress (Vandenkoornhuyse et al. 2015).

Geophysical conditions are well-established drivers of plant and animal distributions, shaping species assemblages through temperature, moisture availability, and resource gradients across landscapes (Amatulli et al. 2018; Seibert et al. 2007; Lausch et al. 2019). These same forces also structure microbial communities (Li et al. 2022; Rodriguez et al. 2022), yet their role in shaping the fungal communities inhabiting the forest canopies, remains poorly understood. The forest canopy is an extreme environment where elevation, solar radiation, and topographic variation create distinct microclimatic conditions, altering moisture retention, UV exposure, and temperature fluctuations—factors known to affect fungal colonization and community composition (Ren et al. 2018; Hughes et al. 2003; Linacre 1982; Körner 2007; Bale et al. 1998; Stage and Salas 2007; Debray et al. 2022). Unlike the forest floor, where soil buffers against environmental extremes, the canopy is directly exposed to these geophysical gradients, potentially making canopy fungi more responsive to subtle shifts in abiotic conditions. Examining how these factors influence the canopy mycobiome is therefore critical for understanding the environmental constraints and ecological processes that govern fungal diversity and composition in forest stands.

In addition to geophysical factors, the canopy mycobiome is also influenced by its close associations with host trees. Significant variation in the canopy microbiome is observed both between tree species (interspecifically) (Siegenthaler et al. 2024) and within a species (intraspecifically) (Xu et al. 2022; Unterseher et al. 2016). Given that temperate European forests are dominated by a few key tree species (Simons et al. 2021), it is crucial to study the canopy microbiome by considering both interspecific and intraspecific variation, as well as forest stand characteristics (Duan et al. 2024). For instance, the leaf area index, which measures the total surface area of leaves per unit of ground area, is directly related to the light distribution in a forest stand and many vegetation functions (Parker 2020). Other host-related factors, such as canopy water content, chlorophyll content, and nutrient levels, vary across forest stands and are key drivers of the leaf mycobiome. Canopy water content, which is heavily influenced by soil water availability, with lowered values usually indicative of forest drought stress, has been shown to impact the canopy mycobiome diversity (Yadav et al. 2005) and the abundance of different fungal functional guilds (Pajares-Murgó et al. 2023). Another crucial indicator of tree stress is canopy chlorophyll content which can, among other biotic and abiotic stressors, signal fungal pathogen infection (Guidi et al. 2007; El Omari et al. 2001). Besides water content and chlorophyll content, canopy nutrients can be another critical driving variable in microbial community assembly as phyllosphere can be an oligotrophic environment for microorganisms. For example, phosphorus, an essential nutrient in the leaf substrate, has been suggested to be a limiting variable for fungal diversity (Sun et al. 2023; Kembel and Mueller 2014).

Although the canopy mycobiome is integral to forest ecosystem function, the environmental and host-driven factors that shape its spatial variability remain poorly understood. Unlike the well-characterized soil and root fungal communities, the canopy—marked by rapid microclimatic fluctuations and complex host-fungal interactions—represents a distinct ecological niche whose underlying drivers have yet to be fully elucidated. In this study, we examine how selected geophysical conditions and host traits explain intraspecific variation in canopy fungal communities in temperate *Fagus sylvatica* (European beech) and *Picea abies* (Norway spruce) forest stands in Bavarian Forest National Park, Germany. We hypothesize that a suite of selected geophysical variables—elevation, solar radiation, topographic position, and topographic wetness index, along with a suite of host-related factors—leaf water content, leaf chlorophyll, leaf phosphorus, and Leaf Area Index, contribute to canopy fungal community composition and diversity. By integrating microbial and landscape-scale perspectives, this study advances our understanding of the ecological processes shaping fungal communities in forest canopies.

## Experimental procedures

### Study site and sampling design

The Bavarian Forest National Park is located along the border between Germany and the Czech Republic. Encompassing an area of about 250 km^2^, the Bavarian Forest National Park together with the neighboring Sumava National Park and the surrounding protected areas, forms the largest contiguous forested areas in Central Europe, the Bohemian Forest Ecosystem that spans approximately 4000 km^2^ (Heurich et al. 2010; van der Knaap et al. 2020). Elevations in the park range from 650 to 1453 m above sea level. The park's vegetation is predominantly forested, covering 97% of the area. The dominant forest tree species include European beech (*Fagus sylvatica*) and Norway spruce (*Picea abies*) (van der Knaap et al. 2020). The park's management strategy, particularly the non-intervention policy adopted in 1983, aims to facilitate the recovery of natural forest dynamics and diversity, providing a site of extensive biodiversity, forestry, and remote sensing research (Latifi et al. 2021; van der Knaap et al. 2020).

We sampled 40 beech and 37 spruce stands from May to July in 2020 and 2021 (Supplementary Table 1). Each forest stand had a size of 30 m × 30 m and dominated by (≥ 75% canopy coverage) one tree species of either beech or spruce. The center of each stand was measured using Differential Global Positioning System (DGPS) Leica GPS 1200 (Leica Geosystems AG, Heerbrugg, Switzerland) with an accuracy of better than 1 m after post-processing. The stand-based sampling design followed a stratified random design over elevation gradient and forest stand age (Duan et al. 2024). For each stand, two trees of the dominant tree species were sampled. Leaf samples were collected from the top canopy using a crossbow and sling shot. Further details about the collection of leaf samples can be found in (Duan et al. 2024).

### Stand geophysical conditions and host traits

Geophysical variables collected included elevation, topographic wetness index (TWI), solar radiation, and topographic position index (TPI). Host factors obtained included leaf area index (LAI), leaf chlorophyll content, leaf phosphorus content, and leaf water content. High-resolution 5-m digital elevation model (DEM) data was used to calculate and generate the stand geophysical conditions based on LIDAR data obtained during a flight campaign in August 2016. See (Zhu et al. 2020) for details on LIDAR collection and processing.

Using the DEM data, we generated TPI, TWI, and solar radiation data in ArcMap 10.8.2. The TPI was calculated using the approach proposed by (Weiss 2001), with the same resolution as the DEM (i.e., 5 m × 5 m cell size). Positive TPI values represent locations higher than the average of their surroundings (ridges), whereas negative TPI values represent locations lower than their surroundings (valleys). TPI values near zero indicate either flat areas (where the slope is near zero) or areas of constant slope (where the point's slope is significantly greater than zero) (Wilson and Gallant 2000). TWI was generated to serve as a proxy for soil moisture (Kopecký et al. 2021). Finally, the solar radiation data was calculated following (Fu and Rich 2002). Elevation, TPI, TWI, and solar radiation values were then extracted for each 30 by 30-m stand using all relevant pixels within the plot, considering the 5-m pixel size of the DEM data.

Stand-scale host traits measured included Leaf Area Index (LAI), stand-average leaf chlorophyll content, stand-average leaf water content and stand-average phosphorus content. The LAI was measured in each forest stand using a plant canopy analyzer (LI-COR LAI-2000) (Darvishzadeh et al. 2008). Other stand-scale host traits were derived by averaging per stand from measurements of two individual trees that were always of the same tree species. For each individual tree, leaf water content, leaf chlorophyll content, and leaf phosphorus content were obtained from leaves pooled per tree and processed in the lab through oven-drying, UV–VIS, and ICP/OES, respectively, as previously described (Duan et al. 2024).

### DNA metabarcoding

For each tree, 0.1 g of leaf material was pooled from a composite of ten leaves or needle branches. A sterile paper hole puncher (0.6 cm Ø) was used to obtain leaf disks from broadleaf samples. To preserve both epiphytic and endophytic fungal communities, samples were not surface sterilized and were homogenized using a Benchmark Beadbug™ Mini Homogenizer (D1030). DNA was extracted using the Qiagen DNeasy Plant Pro Kit and processed with the Qiagen Qiacube Connect, following the manufacturer’s protocol. Samples were processed in batches of 22 to ensure consistency. DNA concentration was quantified using the Quant-iT PicoGreen dsDNA Assay Kit and a Biotek Synergy HTX Multi-Mode Reader, and extracts were normalized to 5 ng/µl, except for samples with lower concentrations (Duan et al. 2024). DNA extracts were normalized to 5 ng/ml, except for samples with a lower concentration. Fungal DNA was amplified using the ITS86 / ITS4-ngs primer set (Tedersoo et al. 2014; Turenne et al. 1999). Polymerase chain reaction (PCR) protocols can be found in the supplementary materials (Supplementary Tables 2 and 3). To mitigate DNA contamination (Welsh and Eisenhofer 2024), we employed the following strategies: (1) one negative extraction control per 23 DNA extractions, (2) two negative PCR controls per 96-well plate, and (3) three spike-in positive controls (Supplementary Table 2) per 96-well plate to control for sample cross-contamination and tag-switching. Each type of control was pooled and sequenced separately. Library preparation and amplicon sequencing (Illumina NovaSeq 6000 SP platform with kit PE250) were performed by Genome Quebec (Montreal, Canada). During library preparation, the Fluidigm Access Array System (Fluidigm, South San Francisco, CA) was utilized for multiplexing, employing CS1 as the forward primer and CS2 as the reverse primer. An indexing PCR with 15 cycles was then conducted to attach indexes and i5/i7 Illumina adapter sequences to the amplicons.

### Bioinformatic and data analyses

Bioinformatic analyses were done in QIIME 2™ (Bolyen et al. 2019) and all data analyses were done in R version 4.2.3 (https://www.R-project.org/). Amplicon Sequence Variants (ASVs), as determined by DADA2 integrated within Qiime2 (v. 2021.8.0), were used to retain a high taxonomic resolution and to improve reproducibility and comparability across datasets (Joos et al. 2020; Callahan et al. 2016). LULU was employed for post-clustering curation to refine ASVs by merging potential artifacts to ensure that rare but ecologically relevant ASVs are preserved while reducing noise in the dataset (Froslev et al. 2017). Taxonomy was assigned using a Naive Bayes classifier implemented in QIIME 2, pre-trained on the UNITE database version 8.3 (10.05.2021) with dynamic clustering thresholds (Nilsson et al. 2019; Abarenkov et al. 2021). Reads retained at each bioinformatic step can be found in Supplementary Table 4. Fungal ASVs were further blank corrected, filtered to only contain fungal reads, and corrected for tag-switching as described previously (Siegenthaler et al. 2024). Low-frequency noise (ASVs with ≤ 5 reads) was excluded (Polling et al. 2022). Read depth was rarefied to 37,636 per sample, based on lowest read depth (Duan et al. 2024). To obtain a canopy mycobiome profile for each stand, samples from individual trees were pooled per stand by averaging the number of reads per ASV after rarefaction. This approach was necessary to align the spatial scale of fungal community analyses with that of the geophysical variables, all of which were measured at the stand level (e.g., Leaf Area Index and solar radiation). Pooling samples at this scale minimizes pseudoreplication while ensuring that fungal community variation is assessed in relation to broader environmental gradients.

No multicollinearity was detected for all explanatory variables based on pairwise correlations and variance inflation factors (VIF < 5). Fungal diversity was calculated using package ‘phyloseq’ (McMurdie and Holmes 2013). The R package ‘ggplot2’ (Wickham and Wickham 2016) and ‘microeco’ (Liu et al. 2021) were used for visualization. Linear regression and PERMANOVA were used to test the explanatory power of geophysical conditions and host traits on canopy mycobiome diversity and community composition, respectively. For PERMANOVA (using 9999 permutations), Bray–Curtis dissimilarity of Hellinger-transformed reads was used to represent differences in fungal community composition. Differential abundance analysis was performed at the fungal family level to identify fungal indicators for significant explanatory variables of diversity and composition, with a minimal occurrence threshold of 30% of samples (Nearing et al. 2022), and on centered log ratio transformed read counts data using the ‘ALDEX2’ package (Fernandes et al. 2014).

## Results

### DNA Illumina sequencing and the forest canopy mycobiome

**Fig. 1:**
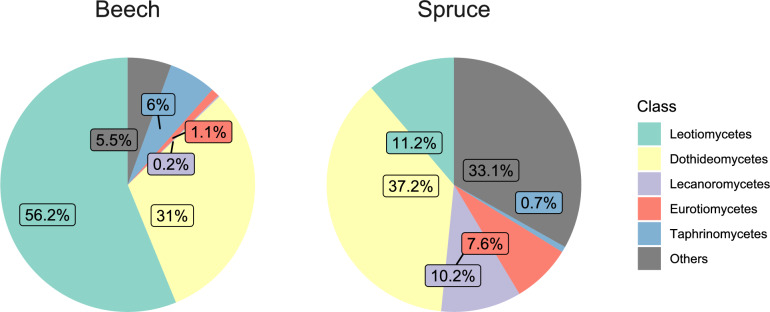
Top five fungal classes in the canopies of beech and spruce forests. Percentages represent relative read abundance

**Fig. 2:**
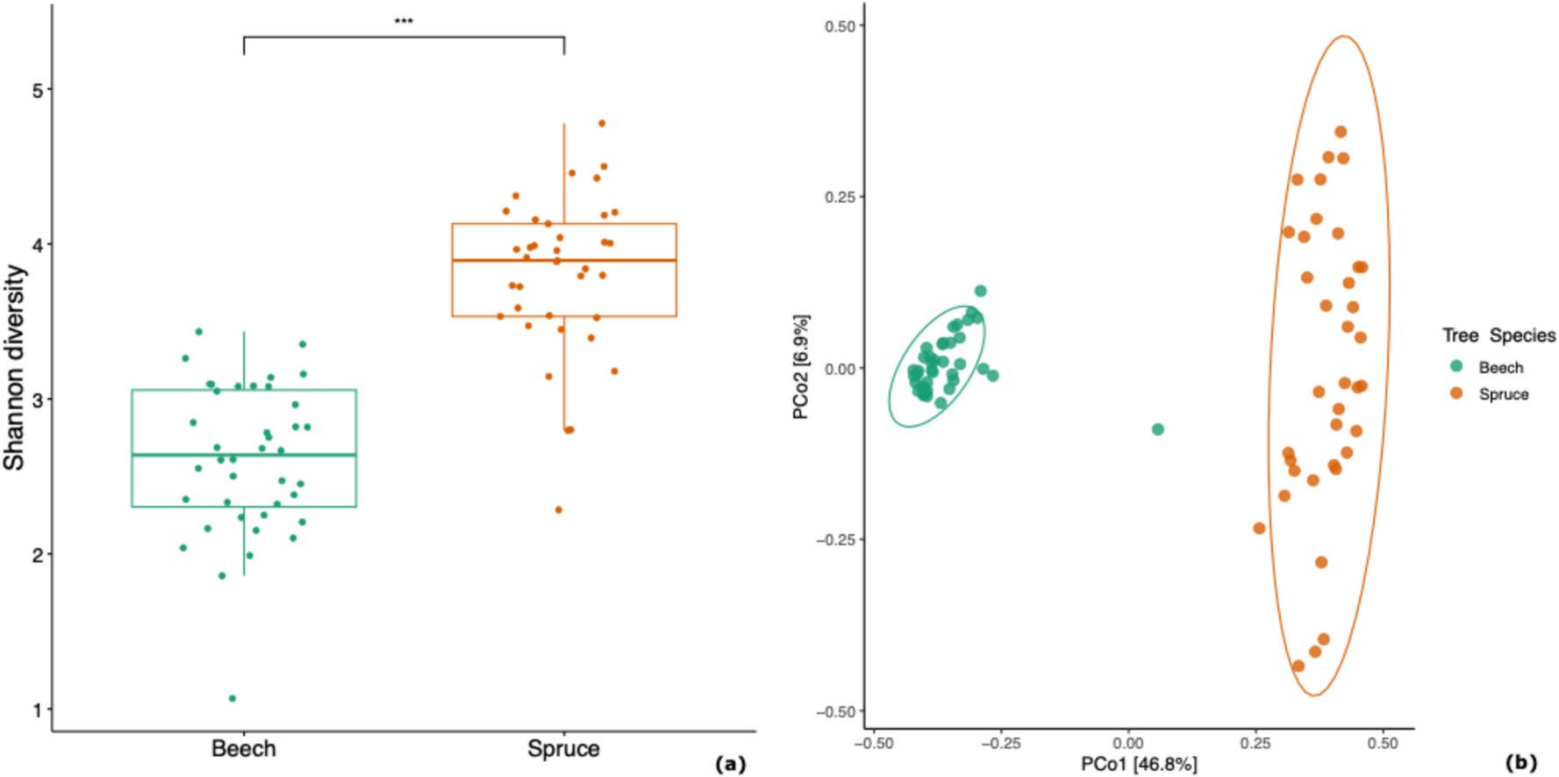
Canopy mycobiome of beech and spruce in Bavarian Forest National Park. (**a**) Variation in Shannon diversity. Rarefied to 37,636 reads per sample. (**b**) Variation in community composition (Bray–Curtis distance)

Across the 77 stand mycobiome profiles remaining after bioinformatic processing, quality filtering, rarefaction, and averaging by forest stand (Supplementary Table 3), 4528 ASVs and 2,897,942 fungal reads were recovered, with an average of 335 ± 126 ASVs (mean ± std) and 37,636 ± 16 reads (mean ± std) per sampled forest stand. These fungal ASVs were from seven phyla, 32 classes, 100 orders, 235 families, and 581 genera. Our results highlighted the distinct and diverse fungal communities associated with beech and spruce canopies in the Bavarian Forest National Park. Notably, beech canopies harbored a higher proportion of *Leotiomycetes* compared to spruce canopies (Fig. 1). As confirmed by PERMANOVA, significant differences in community composition existed between the two host species (Fig. 2), with host tree species accounting for 42% of the observed variation (F(1) = 54.9, *p* < 0.01). In addition, the Shannon diversity of the canopy mycobiome differed significantly between beech and spruce stands (χ^2^(1) = 46.94, *p* < 0.01; Fig. 2a).

### Limited influence of geophysical conditions and host traits on forest canopy mycobiome

The only geophysical variable tested significantly influenced the canopy mycobiome community composition in both beech and spruce stands was elevation. However, its impact was more pronounced in beech stands. Specifically, elevation accounted for 12% of the variation in the canopy mycobiome community composition in beech stands and 8% in spruce stands (Table 1). Additionally, lower elevations were associated with higher canopy fungal Shannon diversity in beech stands (Table 2) but showed no significant association in spruce stands (F(4, 29) = 1.96, Adj.R^2^ = 0.10, *p* = 0.13).

**Table 1:** Geophysical conditions and host traits’ effects on forest canopy mycobiome composition

		Geophysical conditions					Host traits			
		Df	R^2^	F	p-value		Df	R^2^	F	p-value
Beech	Elevation	1	0.12	5.25	< 0.01	Water content	1	0.13	5.60	< 0.01
	TPI	1	0.03	1.10	0.31	P	1	0.06	2.48	0.01
	TWI	1	0.02	0.84	0.60	Chlorophyll	1	0.02	0.87	0.55
	Solar radiation	1	0.02	0.68	0.84	LAI	1	0.03	1.26	0.19
	Residual	34	0.78			Residual	34	0.76		
	Total	38	1.00			Total	38	1.00		
Spruce		Df	R^2^	F	p-value		Df	R^2^	F	p-value
	Elevation	1	0.08	3.06	< 0.01	Water content	1	0.08	3.02	< 0.01
	TPI	1	0.04	1.50	0.08	Chlorophyll	1	0.04	1.67	0.03
	TWI	1	0.03	0.99	0.42	P	1	0.03	1.09	0.31
	Solar radiation	1	0.02	0.82	0.70	LAI	1	0.03	1.29	0.15
	Residual	29	0.79			Residual	30	0.79		
	Total	33	1.00			Total	34	1.00		

Variation partition using PERMANOVA on Bray–Curtis distance on Hellinger-transformed reads. Testing was done “by margin”, using 9999 permutations

**Table 2:** Geophysical conditions on forest canopy mycobiome Shannon diversity of beech (a) and host traits’ effects on forest canopy mycobiome Shannon diversity of spruce stands (b) in Bavarian Forest National Park, based on linear regression

a					
		Geophysical conditions			
		Coefficient	Std.Error	t	p-value
Beech	(Intercept)	5.54	1.99	2.79	0.01
	Elevation	−0.002	0.00	−2.65	0.01
	TPI	0.00	0.01	0.08	0.94
	TWI	−0.07	0.05	−1.50	0.14
	Solar radiation	0.00	0.00	−0.37	0.71
		F(4, 34) = 3.12, Adj.R^2^ = 0.18, *p* < 0.05			

b					
		Host traits			
		Coefficient	Std.Error	t	p-value
Spruce	(Intercept)	2.18	0.62	3.52	< 0.01
	Water content	3.04	0.75	4.05	< 0.01
	Leaf P	0.00	0.00	−0.65	0.52
	Chlorophyll	0.00	0.01	0.48	0.64
	LAI	0.06	0.09	0.66	0.52
		F(4, 30) = 5.79, Adj.R^2^ = 0.36, *p* < 0.01			

Samples have been rarefied to 37,636 reads/sample. Linear regression models on the effect of geophysical conditions on spruce stands and host traits’ effects on beech stands have been omitted since the overall models did not significantly describe the variation in the data (*p* = 0.13 and *p* = 0.26, respectively)

Host traits tested that significantly influenced the canopy mycobiome of beech and spruce were leaf water content, leaf phosphorus, and leaf chlorophyll. Leaf water content emerged as the most significant host trait influencing the canopy mycobiome in both beech and spruce stands. It accounted for 13% of the variation in the canopy mycobiome community composition in beech stands and 8% in spruce stands (Table 1). Additionally in spruce stands, higher leaf water content was associated with higher Shannon diversity (Table 2b). In contrast, no significant association was observed in beech stands (F(4, 34) = 1.40, Adj.R^2^ = 0.04, *p* = 0.26). Besides leaf water content, leaf phosphorus also explained 6% of the variation in canopy mycobiome community composition in beech. While leaf chlorophyll content also explained 4% of the variation in canopy mycobiome community composition in spruce (Table 1).

### Fungal indicators for elevational gradient and canopy water content

We identified 41 fungal families as indicators for specific elevational conditions and canopy water content in beech and spruce forest canopies. Seventeen fungal families in beech stands and nine fungal families in spruce stands were differentially abundant along the elevational gradient (Table 3). For canopy water content, we identified nine fungal families in beech stands and six fungal families in spruce stands to be the indicators for leaf water content level (Table 3).

**Table 3:** Significant differentially abundant fungal families in forest canopies identified by ALDEX2. (aldex.corr, and occurring in at least 30% of the samples). Mean relative abundance was calculated across stands of the same tree species

Stands	Variable	Phylum	Class	Order	Family	Spearman rank correlation rho	Mean Relative abundance
Beech	Elevation	*Ascomycota*	*Dothideomycetes*	*Pleosporales*	*Didymellaceae*	0.68	0.0060
		*Ascomycota*	*Dothideomycetes*	*Pleosporales*	*Pleosporaceae*	0.62	0.0011
		*Ascomycota*	*Lecanoromycetes*	*Ostropales*	*Stictidaceae*	0.62	0.0001
		*Ascomycota*	*Lecanoromycetes*	*Lecanorales*	*Lecanoraceae*	0.61	0.0009
		*Ascomycota*	*Leotiomycetes*	*Helotiales*	*Sclerotiniaceae*	0.60	0.0002
		*Ascomycota*	*Dothideomycetes*	*Pleosporales*	*Didymosphaeriaceae*	0.59	0.0011
		*Ascomycota*	*Taphrinomycetes*	*Taphrinales*	*Taphrinaceae*	0.58	0.0600
		*Ascomycota*	*Dothideomycetes*	*Pleosporales*	*Massarinaceae*	0.53	0.0012
		*Ascomycota*	*Arthoniomycetes*	*Lichenostigmatales*	*Phaeococcomycetaceae*	0.44	0.0005
		*Basidiomycota*	*Microbotryomycetes*	*unidentified*	*Chrysozymaceae*	−0.42	0.0003
		*Basidiomycota*	*Tremellomycetes*	*Tremellales*	*Phaeotremellaceae*	−0.44	0.0010
		*Ascomycota*	*Leotiomycetes*	*Helotiales*	*Helotiaceae*	−0.49	0.0015
		*Ascomycota*	*Leotiomycetes*	*Helotiales*	*Hyaloscyphaceae*	−0.55	0.0040
		*Basidiomycota*	*Exobasidiomycetes*	*Exobasidiales*	*Exobasidiaceae*	−0.55	0.0008
		*Ascomycota*	*Eurotiomycetes*	*Chaetothyriales*	*Trichomeriaceae*	−0.56	0.0060
		*Ascomycota*	*Dothideomycetes*	*Capnodiales*	*Teratosphaeriaceae*	−0.56	0.0078
		*Basidiomycota*	*Tremellomycetes*	*Filobasidiales*	*Filobasidiaceae*	−0.65	0.0045
Beech	Water	*Basidiomycota*	*Agaricomycetes*	*Russulales*	*Russulaceae*	0.56	0.0001
	content	*Basidiomycota*	*Agaricomycetes*	*Boletales*	*Boletaceae*	0.53	0.0001
		*Basidiomycota*	*Tremellomycetes*	*Cystofilobasidiales*	*Mrakiaceae*	0.51	0.0011
		*Basidiomycota*	*Tremellomycetes*	*Filobasidiales*	*Filobasidiaceae*	0.49	0.0045
		*Ascomycota*	*Orbiliomycetes*	*Orbiliales*	*Orbiliaceae*	0.41	0.0052
		*Ascomycota*	*Leotiomycetes*	*Helotiales*	*Dermateaceae*	−0.43	0.4284
		*Ascomycota*	*Dothideomycetes*	*Pleosporales*	*Didymellaceae*	−0.43	0.0060
		*Ascomycota*	*Dothideomycetes*	*Pleosporales*	*Massarinaceae*	−0.46	0.0012
		*Ascomycota*	*Taphrinomycetes*	*Taphrinales*	*Taphrinaceae*	−0.56	0.0600
Spruce	Elevation	*Ascomycota*	*Eurotiomycetes*	*Chaetothyriales*	*Epibryaceae*	0.55	0.0015
		*Basidiomycota*	*Cystobasidiomycetes*	*unidentified*	*Symmetrosporaceae*	0.52	0.0001
		*Ascomycota*	*Dothideomycetes*	*Pleosporales*	*Melanommataceae*	0.48	0.0013
		*Ascomycota*	*Orbiliomycetes*	*Orbiliales*	*Orbiliaceae*	0.46	0.0144
		*Ascomycota*	*Leotiomycetes*	*Helotiales*	*Leotiaceae*	−0.49	0.0009
		*Basidiomycota*	*Agaricostilbomycetes*	*Agaricostilbales*	*Ruineniaceae*	−0.50	0.0001
		*Ascomycota*	*Arthoniomycetes*	*Arthoniales*	*Roccellaceae*	−0.56	0.0002
		*Basidiomycota*	*Tremellomycetes*	*Tremellales*	*Cuniculitremaceae*	−0.66	0.0020
		*Ascomycota*	*Eurotiomycetes*	*Chaetothyriales*	*Cyphellophoraceae*	−0.71	0.0021
Spruce	Water	*Basidiomycota*	*Tremellomycetes*	*Tremellales*	*Bulleribasidiaceae*	0.62	0.0015
	content	*Ascomycota*	*Eurotiomycetes*	*Chaetothyriales*	*Trichomeriaceae*	0.61	0.0072
		*Basidiomycota*	*Tremellomycetes*	*Filobasidiales*	*Filobasidiaceae*	0.49	0.0001
		*Ascomycota*	*Dothideomycetes*	*Capnodiales*	*Teratosphaeriaceae*	−0.48	0.1038
		*Ascomycota*	*Taphrinomycetes*	*Taphrinales*	*Taphrinaceae*	−0.48	0.0072
		*Ascomycota*	*Lecanoromycetes*	*Lecanorales*	*Lecanoraceae*	−0.57	0.0153

## Discussion

Our study demonstrated that geophysical conditions and host traits, specifically elevation and leaf water content, were closely associated with the canopy mycobiome of beech and spruce stands in Bavarian Forest National Park. We identified 41 fungal indicators for specific altitudinal and canopy water conditions, demonstrating the sensitivity of these taxa to these environmental conditions.

### Influence of elevation on canopy mycobiome diversity and composition

Elevation had a significant, albeit limited, impact on shaping the canopy mycobiome in both beech and spruce stands. Lower fungal diversity was observed at higher elevations in beech canopies but not spruce canopies. This suggests that the effect of elevation on phyllosphere fungal communities may be host-specific. One plausible explanation is that beech, as a deciduous broadleaf species typically found at lower elevations, experiences increased environmental stress at higher altitudes (Cordier et al. 2012). In such environment, its broad leaves provide less effective protection against extremes—such as lower temperatures and reduced moisture—thereby reducing fungal diversity, whereas spruce needles, with their more robust structure, better buffer the phyllosphere from such environmental fluctuations (Yuan et al. 2023). These host-specific differences in leaf morphology and the consequent microhabitat conditions likely contribute to the contrasting patterns in fungal community structure observed in beech and spruce canopies, highlighting the importance of considering tree-specific traits when evaluating how environmental gradients shape canopy mycobiomes.

Elevation also corresponded to variation in canopy fungal community composition in both beech and spruce canopies, as evidenced by the changes in the relative abundance of different fungal families along the elevational gradient. This could be due to that canopy fungi have specialized growth requirements that often depend on the presence and condition of their host plants or associated flora along the elevational gradient. For example in beech stands, among the fungal families whose relative abundance increased with elevation, several were known plant pathogenic fungal families (e.g., *Sclerotiniaceae* (Badet et al. 2015; Ma et al. 2019; Navaud et al. 2018) and *Taphrinaceae* (Tsai et al. 2014)). This may be explained by the susceptibility of beech— a deciduous broadleaf species—to potential environmental stress at higher elevations, which can compromise host defenses and facilitate pathogen colonization (Cordier et al. 2012). However, the underlying mechanisms of fungal plant pathogen altitudinal distribution may be more nuanced, requiring further investigation. Similarly, elevational distribution patterns were also observed among certain epifoliar and lichenized fungal families in spruce stands. For instance, *Epibryaceae*, known to be an epifoliar fungi typically exist as bryophyte parasites (Marasinghe et al. 2023), exhibited higher relative abundance at higher elevations. This potentially suggests that these fungi thrive in the cooler, more humid conditions found at these altitudes, or the abundance of bryophyte hosts at higher elevations. Conversely, *Roccellaceae*, a primarily lichenized fungal family (Tehler and Irestedt 2007), were more prevalent at lower elevations in spruce canopies. The environmental conditions at lower elevations, which may include warmer temperatures and different moisture levels, seem to favor the growth of these lichenized fungi, potentially due to the abundance of their lichen symbionts. Since many canopy fungal taxa exhibiting elevational distribution patterns are known to form close associations with host plants or their epiphytic partners, this highlights the unique dependency of canopy fungi on the distribution of their host and partner plants. In contrast to soil fungi—where elevational effects are primarily attributed to variations in abiotic soil properties (Li et al. 2022)—canopy mycobiomes are shaped by both geophysical gradients and the distribution of closely associated flora. These findings reinforce that geophysical conditions, as foundational forces in forests, shape not only the distributions of plants (Asner et al. 2016; Mod et al. 2016) and animals (Porter et al. 2002), but also the canopy mycobiome, advancing our understanding of forest biodiversity and species distribution.

Notably, other investigated geophysical variables—solar radiation, topographic position, and topographic wetness index—did not exhibit significant relationships with canopy fungal diversity or composition at the landscape scale in our study. This may be due to their relatively weak effects or because they operate at different spatial scales.

### Leaf water content links forest stress to forest canopy mycobiome diversity and composition

Leaf water content significantly influenced the canopy mycobiome in both beech and spruce stands in Bavarian Forest National Park. Reduced fungal diversity was observed in drier spruce canopies but not beech canopies. This suggests that the effect of leaf water content on fungal diversity may be host-specific. In spruce, lower needle water content likely lead to altered leaf surface properties—such as reduced wettability and moisture retention (Krupková et al. 2019)—that reduce nutrients availability and constrain the colonization of moisture-sensitive fungal species, leading to lower diversity. Conversely, the relatively permeable surfaces of beech leaves, particularly in the upper canopy (Bahamonde et al. 2018), may support a diverse fungal community even under drier conditions by still allowing nutrients acquisition from the leaf substrates. This observed differential response point to the potential role of leaf surface characteristics in shaping fungal community assembly.

Variation in community composition correlated with canopy water content was observed in both beech and spruce stands, underscoring the close associations between canopy fungi and their host trees. In particular, canopies with reduced leaf water content were enriched in several plant-pathogenic fungal families—such as *Teratosphaeriaceae* (Prihatini et al. 2015; Pérez et al. 2009) and *Taphrinaceae* (Tsai et al. 2014)—suggesting that drier canopy conditions may favor the proliferation of pathogenic fungi. This observation supports the potential role of canopy water content as a mediator of tree stress and increased susceptibility to pathogen colonization (Vannini and Valentini 1994). Canopy water content is recognized as a critical indicator of tree vitality (Konings et al. 2021) and serves as a proxy for water stress (Lyons et al. 2021). And trees with reduced water availability likely shift to a conservative growth strategy, reallocating resources away from growth and defense toward maintenance, which can compromise their resistance to pathogen invasion (Gomez-Gallego et al. 2022). However, it is important to note that canopy water content does not necessarily scale linearly with overall water availability or drought stress, as trees employ distinct adaptive strategies (Rötzer et al. 2017). For example, beech is known to exhibit drought hardening, maintaining or even increasing its leaf water content during the initial stages of drought (Yang et al. 2022), whereas spruce, characterized by reduced plasticity in water management and a lower internal water reserve, may experience more pronounced reductions in water content under similar conditions (Pretzsch et al. 2020; Vornam et al. 2003). Consequently, the underlying mechanism of the observed relationship between canopy water content and fungal community composition should be interpreted in a context-dependent manner, considering tree growth strategies and environmental conditions.

Two other host factors—canopy chlorophyll and phosphorus were also important drivers of the canopy mycobiome composition of spruce and beech stands, respectively. While chlorophyll is generally a universal indicator of photosynthetic activity and tree health (Guidi et al. 2007; El Omari et al. 2001), in spruce it may be particularly informative of needle physiology—affecting the production of photosynthates and defensive compounds (Agathokleous et al. 2020)—which in turn modulates the microenvironment on the needle surface and influences fungal colonization. In contrast, the broad leaves of beech have higher nutrient content (Wright et al. 2004), so variations in phosphorus availability can directly alter the nutrient profile of the leaves, thereby modulating canopy fungal community composition (Sun et al. 2023).

### Enhancing forest monitoring with eDNA from the top canopy

Environmental challenges like droughts can significantly alter the forest canopy mycobiome by influencing tree vitality (Peñuelas et al. 2012). Beech and spruce stands in BFNP face a suite of stressors, such as a warmer and drier climate (Martinez del Castillo et al. 2022; Einzmann et al. 2021). Additionally, the spruce dominated stands are prone to bark beetle outbreaks (König et al. 2023). These environmental stressors compromise tree vitality, which is evident through indicators like loss of leaf water potential, defoliation, and crown dieback (Walthert et al. 2021). Further exploring the relationships between these tree vitality indicators and the canopy mycobiome can enhance our understanding of how environmental stressors impact forest ecosystems.

Our study offers insights into this rare biome by exploring the role of geophysical conditions and host traits. Given that many geophysical and vegetation traits can be derived from remote sensing data (Skidmore et al. 2021, 2022), integrating eDNA analyses as employed in this study with remote sensing holds promise for advancing our understanding of forest canopy microbial biodiversity and species distribution, bridging the gap in monitoring Earth’s major ecosystems through an eDNA approach (Cambon et al. 2023). Overall, our work contributes to a more nuanced perspective on the spatial organization of top canopy mycobiomes, thereby deepening our understanding of this critical forest biome.

## Conclusions

We examined how geophysical conditions (elevation, solar radiation, topographic position and wetness) and host traits (canopy water content, canopy chlorophyll, canopy phosphorus, and leaf area index) explain intraspecific variation in the canopy mycobiome of *Fagus sylvatica* (European beech) and *Picea abies* (Norway spruce) stands in the Bavarian Forest National Park, Germany. Our results showed that elevation and canopy water content were important drivers of fungal diversity and community composition for both species. While two other host factors—canopy chlorophyll and phosphorus were also important drivers of the canopy mycobiome composition of spruce and beech stands, respectively.

Investigated geophysical variables other than elevation—solar radiation, topographic position, topographic wetness index—did not exhibit significant relationships with canopy fungal diversity or composition at our landscape scale. While these factors may be influential in other contexts or at different scales, our results suggest they were not the primary drivers of forest canopy mycobiome in our study area.

Overall, our findings illustrate how altitudinal variation and host-associated factors shape the canopy mycobiome, shedding light on the spatial patterns of fungal communities in forest canopies. Given that many geophysical conditions and vegetation traits can be retrieved using remote sensing, integrating eDNA analyses, as employed in this study, with remote sensing holds promise for scaling the study of canopy microbial biodiversity and species distributions across broader spatial scales. Future research could further investigate how these key variables interact with additional biotic and abiotic factors to refine our understanding of canopy mycobiome assembly and maintenance in temperate forest ecosystems.

## Supplementary Information

Below is the link to the electronic supplementary material.

Supplementary file1 (DOCX 32 kb)

Supplementary file2 (XLSX 1402 kb)

## Data Availability

The authors declare that the data supporting the findings of this study are available within its Supplementary Information files. Should any raw data files be needed in another format, they are available from the corresponding author upon reasonable request.
