# Supplementary material for: Landscape-scale variation in the canopy mycobiome in temperate beech and spruce forest stands explained by leaf water content and elevation
Source: Eur J For Res. 2025 Mar 29;144(3):443–55. doi: 10.1007/s10342-025-01768-3 (PMC12159088; doi:10.1007/s10342-025-01768-3)
Supplement: Supplementary file 1 — Supplementary file1 (DOCX 32 kb) [file 10342_2025_1768_MOESM1_ESM.docx]

Landscape-scale variation in the canopy mycobiome in temperate beech and spruce forest stands explained by canopy water content and elevation

Yiwei Duan^1*^, Andjin Siegenthaler^1^, Andrew K. Skidmore^1^, Marco Heurich^2^, Haidi Abdullah^1^, Anthony A. Chariton^3^, Ivo Laros^4^, Mélody Rousseau^1^, G. Arjen de Groot^4^

Supplementary Table 1. Sampling design

| Forest stand sampled | 2020 | 2021 |
| --- | --- | --- |
| Beech stands | 28 | 12 |
| Spruce stands | 27 | 10 |

Supplementary Table 2. PCR reagents for the ITS amplifications of leaf DNA extracts

| **Component** | **Supplier** | **Final Concentration** | **Volume (µl)** |
| --- | --- | --- | --- |
| H2O | Sigma-Aldrich | NA | 1.675 |
| PCR buffer | Thermo Fisher | 1x | 1.250 |
| MgCl2 | Thermo Fisher | 2.5 mM | 0.625 |
| Trehalose | Thermo Fisher | 5% | 6.250 |
| Bovine serum albumin | VWR | 200 ng/µl | 0.125 |
| dNTP | VWR | 200 µM each | 0.250 |
| PrimerMix | Biolegio | 250 µM each | 0.125 |
| Platinum Taq Polymerase | Thermo Fisher | 0.08 U/µl | 0.200 |
| Subtotal |  |  | 10.5 |
| DNA template or controls |  |  | 2.0 |
| Total |  |  | 12.5 |
| Primer sequence  (5' to 3')* | ITS86F_CS1: ACACTGACGACATGGTTCTACAGTGAATCATCGAATCTTTGAA  ITS4-ngsR_CS2: TACGGTAGCAGAGACTTGGTCTTCCTSCGCTTATTGATATGC | | |
| * The primer adapter sequences (CS1/CS2) are underlined. | | | |
| Synthetic spike-in sequence (5’ to 3’):  GCAGCACGCGCTGTTCGATCTACCCTACACTCGTGTAATACCCGTTTATGTCGAAACCGTTGCTAAGGCATAAACCAGCTGTAAACGTCTCTTCACGAGATTTCCATTAATCACGCATTAGCTGACGGTGGGTAACTGATGTTAGCCTCTAAATAAGCCACATTACCAGGCTCTCTCCAAAGCGACGGCAGGCACGACATGGATTGTAGAGTT  Note: The sequence was randomly generated in silico using R. The synthetic sequence was the same length as the amplified products and contained no more than three homologous nucleotides. Prior to synthesis, primers were added to the synthetic sequence with the synthetic sequence synthesized by Integrated DNA Technologies, Inc. Sequence: | | | |

Supplementary Table *3*. PCR programs for the ITS amplifications of leaf DNA extracts

| **Step** | **Temperature** | **Time** |
| --- | --- | --- |
| 1 | 94°C | 2 min |
| 2 | 94°C | 30 sec |
| 3 | 62°C | 3 min -1°C/cycle |
| 4 | 72°C | 1 min |
| 5 | Go to Step 2, 14x | |
| 6 | 94°C | 30 sec |
| 7 | 48°C | 3 min |
| 8 | 72°C | 1 min |
| 9 | Go to Step 6, 19x | |
| 10 | 72°C | 10 min |
| 11 | 10°C | For ever |
| 12 | End |  |

Supplementary Table 4. Summary of bioinformatic pipeline for fungal ITS metabarcoding data.

| **Filtering step** | **reads** | **ASVs** | **Samples** |
| --- | --- | --- | --- |
| Raw demultiplexed data | 2020: 155,981,020 (F/R)  2021: 294,382,428 (F/R) |  | 2020: 481  2021: 530 |
| Primer trimming (cutadapt:  Primers trimmed: ITS86F & ITS4-ngsR | 2020: 153,450,121 (F/R)  2021: 286,779,865 (F/R) |  | 2020: 481  2021: 530 |
| Denoising (dada2: trunc-len-f: 0 trunc-len-r: 0, MaxEE = 2) | 2020: 124,873,573  2021: 212,263,734 | 2020: 36,310  2021: 37,748 | 2020: 481  2021: 530 |
| Merge 2020 and 2021 runs  (qiime feature-table merge)  and removal of soil samples not pertinent to this study | 256,251,056 | 43,846 | 700 |
| LULU post clustering curation (84% minimum match, minimum relative co-occurrence: 0.95) | 256,251,056 | 27,778 | 700 |
| Removal of positive control (N=6), Positive-spiked control (N=6) and negative control samples (N=36) | 242,300,931 | 27,409* | 652 |
| Blank correction (Removal criteria: max reads in blank ≥ max reads in samples) | 219,699,327 | 27,230 | 652 |
| Non-fungal read filter | 106,783,418 | 15,584 | 652 |
| Tag-switching (tag-switching threshold: 0.01%)** | 106,428,712 | 15,584 | 652 |
| Final data files | 106,428,712 | 15,584 | 652 |
| Sample selection: Exclusion of samples that were not pertinent to this study | 2,897,942 | 4,528 | 154 |

* 368 ASVs removed that only occurred in the 2020 spiked control samples only. These spiked controls also included soil controls

** based on max positive control reads in samples as fraction of total positive control reads

F/R: Forward and reverse reads, prior to merging pair-end reads
